# Ancestral protein resurrection and engineering opportunities of the mamba aminergic toxins

**DOI:** 10.1038/s41598-017-02953-0

**Published:** 2017-06-02

**Authors:** Guillaume Blanchet, Doria Alili, Adèle Protte, Gregory Upert, Nicolas Gilles, Livia Tepshi, Enrico A. Stura, Gilles Mourier, Denis Servent

**Affiliations:** 1 CEA Institut des Sciences du Vivant Frédéric Joliot, Service d’Ingénierie Moléculaire des Protéines (SIMOPRO), Université Paris-Saclay, Gif-sur-Yvette, Paris, 91190 France; 2 UFR Sciences de la Vie, Université Pierre et Marie Curie (UPMC), Paris, 75005 France

## Abstract

Mamba venoms contain a multiplicity of three-finger fold aminergic toxins known to interact with various α-adrenergic, muscarinic and dopaminergic receptors with different pharmacological profiles. In order to generate novel functions on this structural scaffold and to avoid the daunting task of producing and screening an overwhelming number of variants generated by a classical protein engineering strategy, we accepted the challenge of resurrecting ancestral proteins, likely to have possessed functional properties. This innovative approach that exploits molecular evolution models to efficiently guide protein engineering, has allowed us to generate a small library of six ancestral toxin (AncTx) variants and associate their pharmacological profiles to key functional substitutions. Among these variants, we identified AncTx1 as the most α_1A_-adrenoceptor selective peptide known to date and AncTx5 as the most potent inhibitor of the three α2 adrenoceptor subtypes. Three positions in the ρ-Da1a evolutionary pathway, positions 28, 38 and 43 have been identified as key modulators of the affinities for the α_1_ and α_2C_ adrenoceptor subtypes. Here, we present a first attempt at rational engineering of the aminergic toxins, revealing an epistasis phenomenon.

## Introduction

Evolutionary processes of venomous animals have selected enzymes and disulfide-rich peptides in their venoms to improve their ability to subdue their prey and defend against predators. These compounds act predominantly on few, well characterized and physiologically-relevant molecular targets of the envenomed animals^1^. Recruited by convergent evolution, toxins impact mainly the haemostatic, nervous and cardiovascular systems^2^. Some toxins present in venoms, in spite of their debilitating effects, have become live-saving drugs^3–5^. To exert their biological activities, toxins interact mainly with ion channels, coagulation factors, nicotinic receptors, cell membrane or enzymes but only rarely with the most important class of physiological targets, i.e, the G protein-coupled receptors (GPCRs)^6^. Compared to numerous toxins that act on voltage-gated or ligand-gated ion channels, only a few toxins that interact with GPCRs have been identified. These toxins have been isolated mainly from cone snails venoms such as conopressin interacting with vasopressin receptor^7^, contulakin-G which binds to neurotensin receptor^8^, ρ-conotoxin TIA specific of the α_1A_-adrenoceptor^9^ and the τ-conotoxin CnVA that interacts with the somatostatin sst3 receptor^10^. The black widow spider and the gila monster provide further examples of GPCRs interacting toxins, namely, α-latrotoxin interacting with the latrophilin receptor^11^ and exenatide, exploited as an anti-diabetic drug^12^, that targets the GLP-1 receptor, respectively. In addition, mamba venoms contain aminergic toxins that recognize various bioaminergic receptors^13–19^.

**Figure 1:**
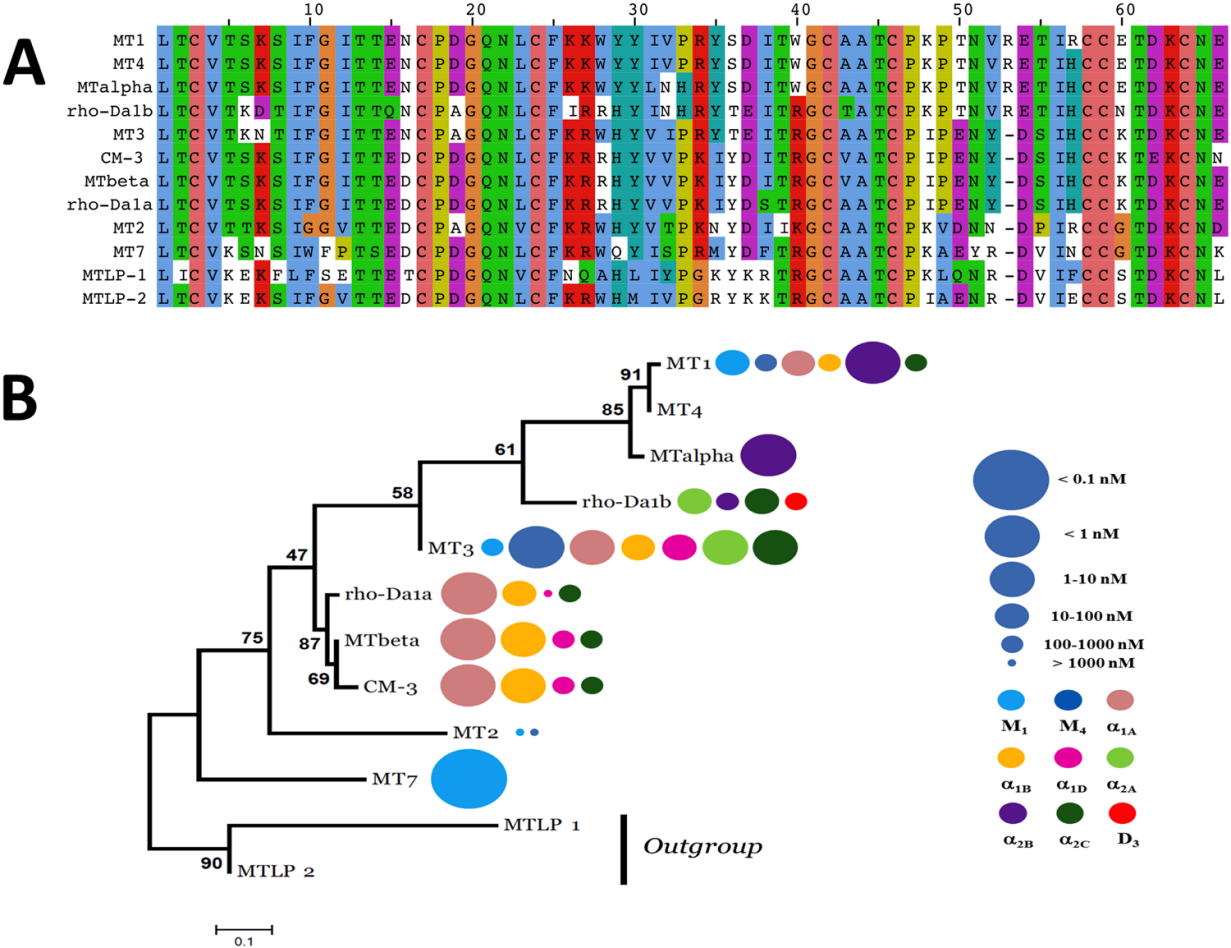
Sequence alignment and phylogenetic tree of aminergic toxins. (**A**) Sequence alignment of the aminergic toxin family. Color display is settled in order to emphasize amino acid conservation along all toxins. (**B**) Maximum likelihood tree and associated function of the aminergic toxins. The best evolutionary model established by MEGA5 was the Dayhoff model using a discrete Gamma distribution (+G). Bootstraps value higher than 50% are shown. MTLP-1 and 2 were used as out-group. Toxin’s functions are also illustrated by circles identifying the receptor targeted (color) and the corresponding K_i_ value (circle size), as described in the right legend, at the right side of each toxin name.

Aminergic toxins belong to the three-finger fold toxin (3FT) superfamily, a structural fold known to support a large diversity of biological functions^20^. Recently, protein engineering using a cDNA display approach was applied to 3FT to create large libraries of randomized sequences. After repeated rounds of expression and screening, interleukin-6 receptor ligands^21^ and serine protease inhibitors^22^ were selected, highlighting the high functional versatility of this structural template. On the same scaffold, a more rational protein engineering was developed using a loop grafting strategy leading to chimeric toxins with new functional profiles on muscarinic and adrenergic receptors^23^. Despite sequence identities that vary from 70 to 98% (Fig. 1A), aminergic toxins display highly variable pharmacological profiles (Fig. 1B). On the one hand, exceptional selectivity is exemplified by MT7 or MTα which interact exclusively with one receptor subtype (Fig. 1B), while on the other hand, MT3 interacts efficiently and non-selectively with seven different aminergic receptors (Fig. 1B). These observations suggest that during the evolution of these toxins various functional properties diverged, were modified and, the least useful ones for the snake, dispensed with. It is possible that toxins with interesting activities on the bioaminergic receptor family might have existed in the past. Given the difficulty of selecting potentially interesting toxins among the vast number of variants that a traditional rational engineering approach might suggest, we decided to test an alternative semi-rational method that involves ancestral protein resurrection. By rewinding the important changes that might have occurred during evolution of a protein family, this approach might be useful to correlate sequence variations with toxin activities within this family.

In the recent years, a growing number of works relating to the use of ancestral protein resurrection have been published to address different issues like understanding protein function evolution^24^, fold stability over time^25^, protein solubility^26^ or to perform protein engineering. By constructing and testing function of these ancestral proteins and comparing the results with extant proteins, this method is able to follow amino acids changes in the evolutionary history and measure their impact on their properties. This “evolutionary biochemistry”^27^ open also a new avenue for protein engineering^28^ by creating small but functionally rich libraries^29^. As the first proof of concept for enzyme engineering, Gaucher and colleagues use the REAP (Reconstructing Evolutionary Adaptive Paths) to engineer new Taq polymerases with original properties^30, 31^.

Here, we use the ancestral protein resurrection methodology on the aminergic toxin group in order to pinpoint the functional substitutions which may have occurred during their evolution and use them for molecular engineering. It is the first application of this methodology on a mini-protein/peptide family. We compare the pharmacological profiles of the natural and six ancestral toxins found at different nodes of the aminergic toxin tree and describe the functional modulations between them that could be associated with key substitutions. A first attempt to engineer more potent aminergic 3FT validates these findings by obtaining the most selective α_1A_ and the most potent α_2_ adrenergic toxins.

## Results

### Aminergic toxins pharmacology, phylogeny, ancestral states reconstruction and structure

**Figure 2:**
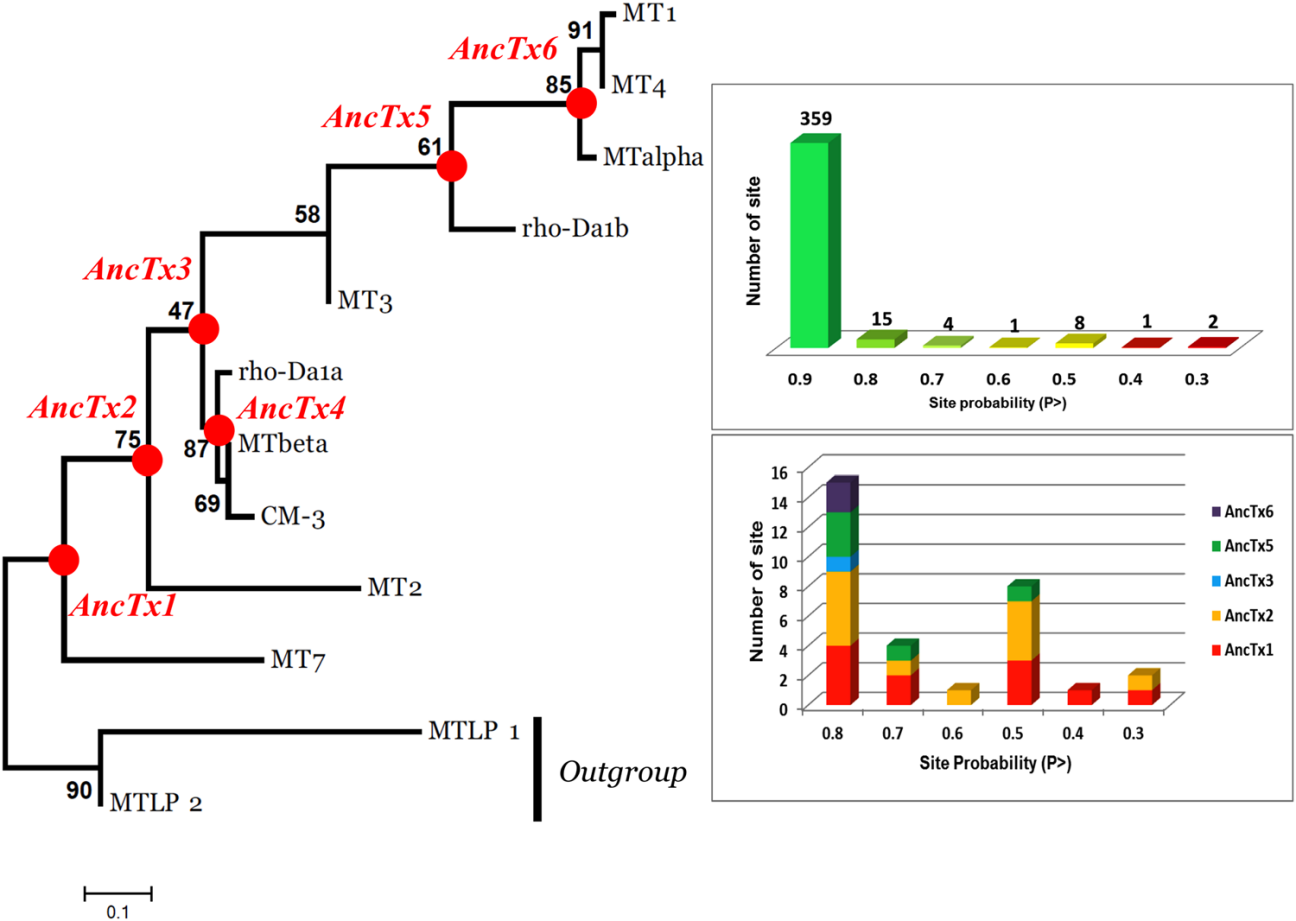
Maximum likelihood tree of the aminergic toxins. The best evolutionary model established by MEGA5 was the Dayhoff model using a discrete Gamma distribution (+G). Bootstraps value higher than 50% are shown. MTLP-1 and 2 were used as out-group. Six ancestral sequences are figured on the nodes of the aminergic toxins tree. Quality of the reconstruction is shown on right side upper panel. For each site, a probability P is given. In total 390 sites were reconstructed. Among them, only 31 showed a P < 0.9, highlighting a good quality of the reconstruction. As shown in the lower panel, a high proportion of these ambiguous sites are presented in the sequences of AncTx1 and 2. The ambiguities were resolved by chosen the residues with the best probability. Due to the high divergence of MT7 and MT2 in term of sequence and function, this choice leads AncTx1 and 2 to be closer of AncTx3 and ρ-Da1a than MT7/MT2.

**Figure 3:**
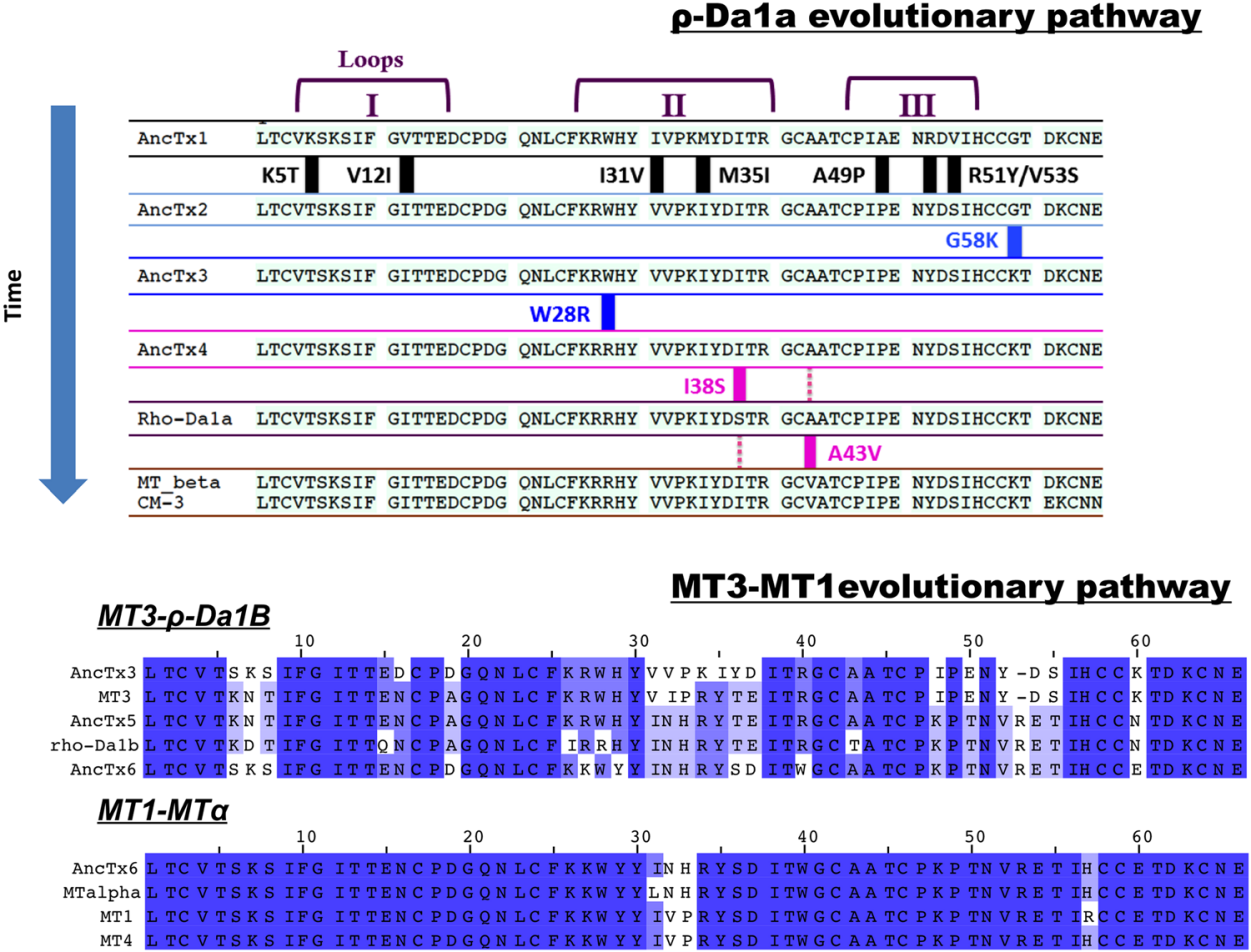
Amino acid substitutions in the aminergic toxins and their ancestors from the two evolutionary pathways. Sequences are following the time/tree topology of the aminergic toxins. MT7 and MT2 sequences are not shown in the ρ-Da1a pathway due to divergences in sequence and function. Amino acid substitutions are highlighted in the first pathway. MT1-MT3 evolutionary pathway is also illustrated. Color display is settled in order to emphasize amino acid conservation along the toxins. Stronger blue color indicates residue conservation among all sequences.

Previously published phylogenetic analysis showed that the aminergic toxins are a functional family specifically developed in the mamba venoms and that a high sequence identity is shared among them (at least 70%, Fig. 1A)^19^. Based on these results, the maximum likelihood (ML) tree of the mamba aminergic toxins was reconstructed (Fig. 1B). The two MTLP 1 and 2 from *Naja kaouthia* were considered as an out group^19^. The ML methodology allows also the reconstruction of the sequences at each nodes of the tree, also called ancestral sequences (Fig. 2). From this reconstruction, six ancestral toxin sequences were derived, and named from AncTx1 to AncTx6. For example, AncTx1 represents the ancestor of MT7 and AncTx2. The six sequences reconstructed are made by 390 sites in total (65 × 6, Fig. 2 - upper and lower panels on the right). On these 390 sites reconstructed, 359 (92%) showed a probability P > 0.9, highlighting a good degree of confidence for the AncTx sequence reconstructions. Among the 31 sites with a P < 0.9, also called ambiguous sites, 15 had a high P value (P > 0.8). It is important to mention that 74% (23/31) of these ambiguous sites were from AncTx1 and 2. These ambiguities were resolved by choosing the most probable residues. Due to the high divergence of MT7 and MT2 in term of sequence and function (Fig. 1), this choice has lead AncTx1 and 2 to be closer to AncTx3 and ρ-Da1a than to MT7/MT2. More precisely, AncTx1 displayed only 7 substitutions spread over the 3 loops compared to AncTx2 (Fig. 3) but as many as 13 modifications compared with MT7. AncTx2 differs by only one substitution (G58K) compared to AncTx3 and by 16 from MT2, most of them located on finger III. A single substitution caused each transition: from AncTx3 to AncTx4 (W28R), from AncTx4 to ρ-Da1a (I38S) and from AncTx4 to MTβ (A43V) (Fig. 3).

On the other hand, 10 substitutions were needed in finger I and II to go from AncTx3 to MT3. AncTx5, at the node of ρ-Da1b, contains 10 substitutions spread over fingers II and III compared to MT3 (including the insertion of valine 52), but only 5 and 7 substitutions spread over fingers I and II compared to ρ-Da1b and AncTx6, respectively. Finally, AncTx6 was close to MTα and MT1. Only one (I31L) and three substitutions (N32V, H33P, H57R) differentiated AncTx6 from MTα, and MT1, respectively (Fig. 3).

The six toxins were synthesized by solid-phase chemical synthesis, refolded and purified by reverse phase HPLC (see materials and methods). The CD spectra of AncTxs showed pronounced maxima and minima at 195 and 215 nm, respectively, typical of β-sheet structure. The CD spectrum of AncTx6 differed slightly from the other AncTxs with additional maxima and minima at 220 nm and 230 nm, that might reflect some local structural variations, as previously observed for MT1^32^ (Supplementary Fig. S1).

**Figure 4:**
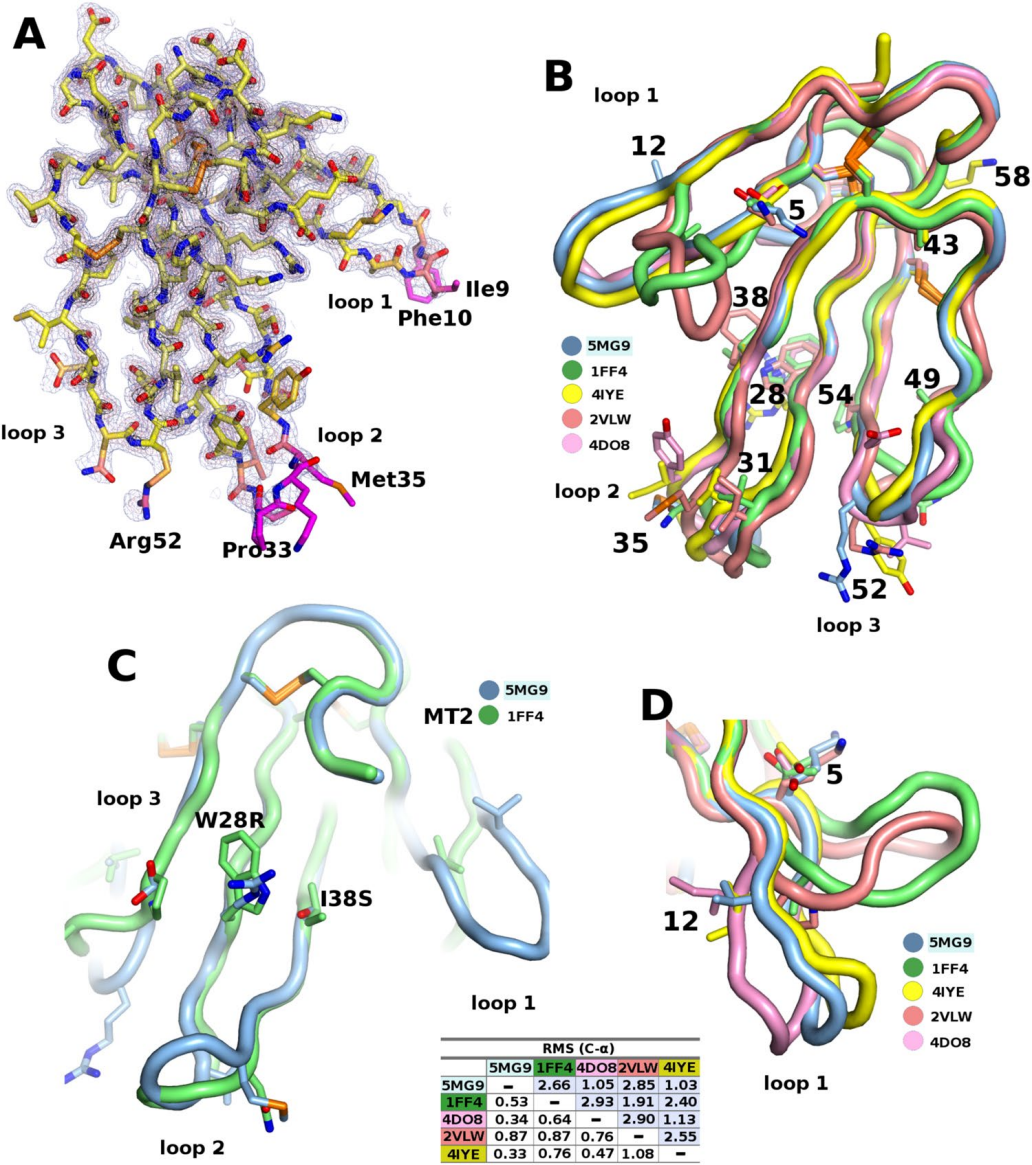
X-ray structure of AncTx1 (variant W28R-I38S). (**A**) Three-finger fold structure of AncTx1 variant. (**B**) superimposition of AncTx1 variant structure with other aminergic toxins from mamba, MT1 (PDB id: 4DO8), MT2 (PDB id: 1FF4), MT7 (PDB id: 2VLW) and ρ-Da1a (PDB id: 4IYE). (**C**) Superimposition of AncTx1W28R-I38S and MT2 loop 2. (**D**) structural variations at the tip of loop 1 of aminergic toxins highlighting a large kink in MT7 and MT2. The RMSD from C-α for core residues (white background) shows that the backbones for all the toxins superimpose well, but the agreement deteriorates if loop residues are also added to the calculation (light blue background).

In order to visually understand the evolution of the AncTxs, the crystallographic structure of AncTx1 (variant W28R-I38S) was solved (Fig. 4, Table S1). As it might have been expected, the structure was rigid, except at the tips of the three loops (Fig. 4A). Superimposition on other known mamba toxins, MT1 (PDB id: 4DO8^23^), MT2 (PDB id: 1FF4), MT7 (PDB id: 2VLW^33^) and ρ-Da1a (PDB id: 4IYE^34^) showed strong conservation of the backbone (Fig. 4B). The RMSD from C-α for core residues (white background) shows that the backbones for all the toxins superimpose well, but the agreement deteriorates if loop residues are also added to the calculation (light blue background) (Table in Fig. 4). AncTx1W28R-I38S represents well AncTx1 as the two mutations on loop 2 do not perturb the structure, as can be verified by comparing AncTx1W28R-I38S with MT2 (Fig. 4C). Loop 1 is more susceptible to backbone variations, and both MT7 and MT2 are subjected to a large kink at the tip of this loop. However loop 1 of MT1, ρ-Da1a and AncTx1W28R-I38S remain straight (Fig. 4D).

From the first screening with 1 and 5 µM of ancestral toxins final concentrations on various known targets of aminergic toxins, the first 4 ancestors (AncTx1 to AncTx4) display similar pharmacological properties on adrenoceptors α_1A_, α_1B_, α_1D_ and α_2C_ as compared to ρ-Da1a, MTβ and CM-3. Despite some minor modulations in the affinity constants of ancestral and actual toxins, the targeted receptors remained the same in this phylogenetic arm. However, for toxins located in the upper part of the phylogenetic tree (MT3, AncTx5, ρ-Da1b, AncTx6, MTα, MT1), the receptor targeted and the selectivity profiles varied widely. Those toxin-receptor couples, that showed a significant radioligand displacement, were further analyzed by dose-response competition experiments in order to characterize precisely their selectivity profiles. The results are separated below along two evolutionary pathways to highlight changes in the affinity profile towards the various receptor subtypes that may have been the targets of the ancestral toxins.

### ρ-Da1a evolutionary pathway

**Figure 5:**
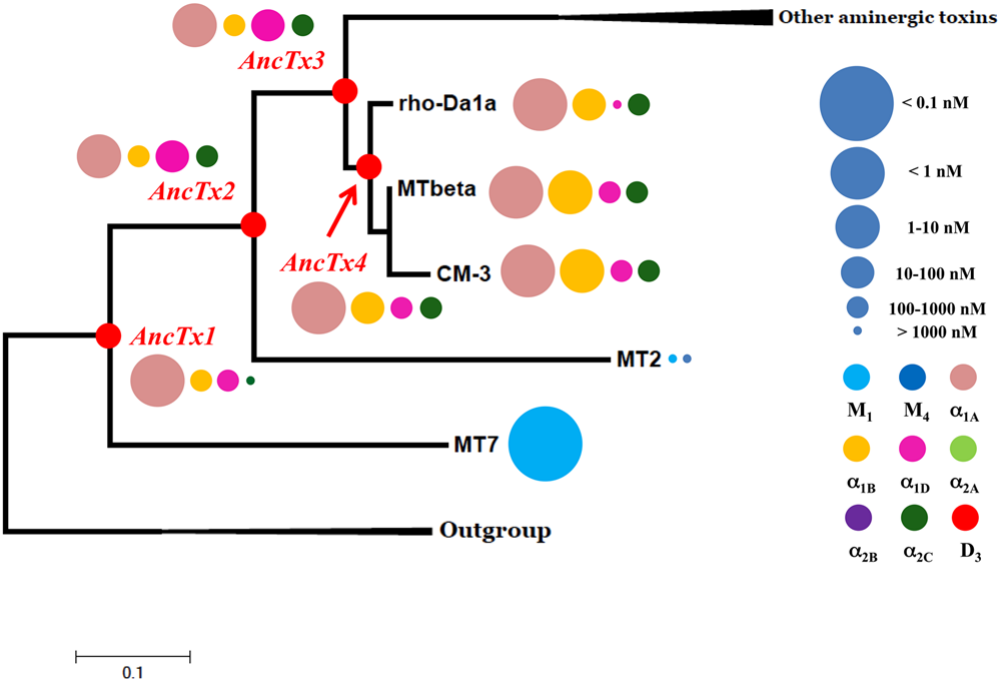
Maximum likelihood tree and associated function of the aminergic toxins from ρ-Da1a evolutionary pathway. Bootstraps value higher than 50% are shown. Toxin’s functions are illustrated by circles identifying the receptor targeted (color) and the corresponding K_i_ value (circle size), as described in the right legend, at the right side of each toxin name.

The ρ-Da1a pathway branches out from four ancestors with a primary structure similar to ρ-Da1a, MTβ and CM-3. These ancestral toxins were able to interact with adrenoceptors subtypes α_1A_, α_1B_, α_1D_ and α_2C_ with affinity constants varying from nanomolar to micromolar range (Supplementary Fig. S2 and Fig. 5). On the α_1A_ receptor, the four reconstructed toxins showed homogeneous pK_i_ values, ranging from 9.62 for AncTx1 to 8.78 for AncTx3 (Table 1). Natural toxins have similar pK_i_: 9.19 for ρ-Da1a and 9.43 for CM-3. Thus, the different substitutions that occurred along this evolutionary tree appear to have had a limited impact on α_1A_ subtype toxin affinity.

**Table 1:** Affinity constant (pKi ± SEM) of ρ-Da1a evolutionary pathway toxins for adrenoceptors α_1A_, α_1B_, α_1D_ and α_2C_.

Receptor subtype Toxins	α_1A_	α_1B_	α_1D_	α_2C_
AncTx1	9,62 ± 0,10	6,03 ± 0,02	6,58 ± 0,05	5,53 ± 0,06
AncTx2	8,79 ± 0,25	6,78 ± 0,05	7,14 ± 0,02	6,34 ± 0,01
AncTx3	8,78 ± 0,18	6,89 ± 0,08	7,27 ± 0,02	6,35 ± 0,04
AncTx4	9,24 ± 0,09	7,60 ± 0,02	6,39 ± 0,07	6,83 ± 0,17
ρ-Da1a^a^	9,19	7,28	5,95	6,85
MTβ^b^	9,30	7,99	6,87	6,77
CM-3^b^	9,43	7,98	6,98	6,78
AncTx1 I38S	9,33 ± 0,01	4,75 ± 0,22	6,44 ± 0,01	4,90 ± 0,19
AncTx1 W28R-I38S	8,59 ± 0,09	6,41 ± 0,04	3,71 ± 0,11	4,85 ± 0,14

a: [19], b: [32].

AncTx1 showed the lowest affinity on the α_1B_ receptor, with a pK_i_ value equals of 6.03. A six-fold increase in affinity characterizes the evolution from AncTx1 to AncTx2 and AncTx3 having respectively a pK_i_ of 6.78 and 6.89. Further gains in affinity for the α_1B_ receptor were measured for AncTx4 (pK_i_ = 7.60) and for the wild-type toxins ρ-Da1a (7.28), CM-3 (7.98) and MTβ (7.99) (Table 1, Supplementary Fig. S2), suggesting that the W28R substitution between AncTx3 and AncTx4 might have been important for the affinity increase observed for this receptor subtype (Fig. 5).

A similar evolutionary pattern, as that observed on α_1B_ receptor, characterizes the pharmacological profiles of the different reconstructed toxins on the α_2C_ receptor subtype. Indeed, AncTx1 interacted with the lowest affinity on the α_2C_ receptor (pK_i_ of 5.53) and a seven-fold gain in affinity occurred with the evolution to AncTx2 and AncTx3 with pK_i_ values of 6.34 and 6.35, respectively. A minor improvement was achieved with AncTx4 that interacted with the α_2C_ subtype with a slightly higher affinity (pK_i_ = 6.83), surpassing its ancestors to reach values comparable to actual toxins: MTβ (pK_i_ = 6.77), CM-3 (pK_i_ = 6.78) and ρ-Da1a (pK_i_ = 6.85) (Table 1, Supplementary Fig. S2).

Regarding the α_1D_ subtype, the pK_i_ values for AncTx2 and AncTx3 (7.14 and 7.28, respectively) were higher compared to AncTx1 (6.58), AncTx4 (6.39) also compared to modern toxins with affinity constants ranging widely from 5.95 for ρ-Da1a to 6.98 for CM-3 (Table 1, Supplementary Fig. S2).

#### Structure-activity relationship inference: case of AncTx4

**Figure 6:**
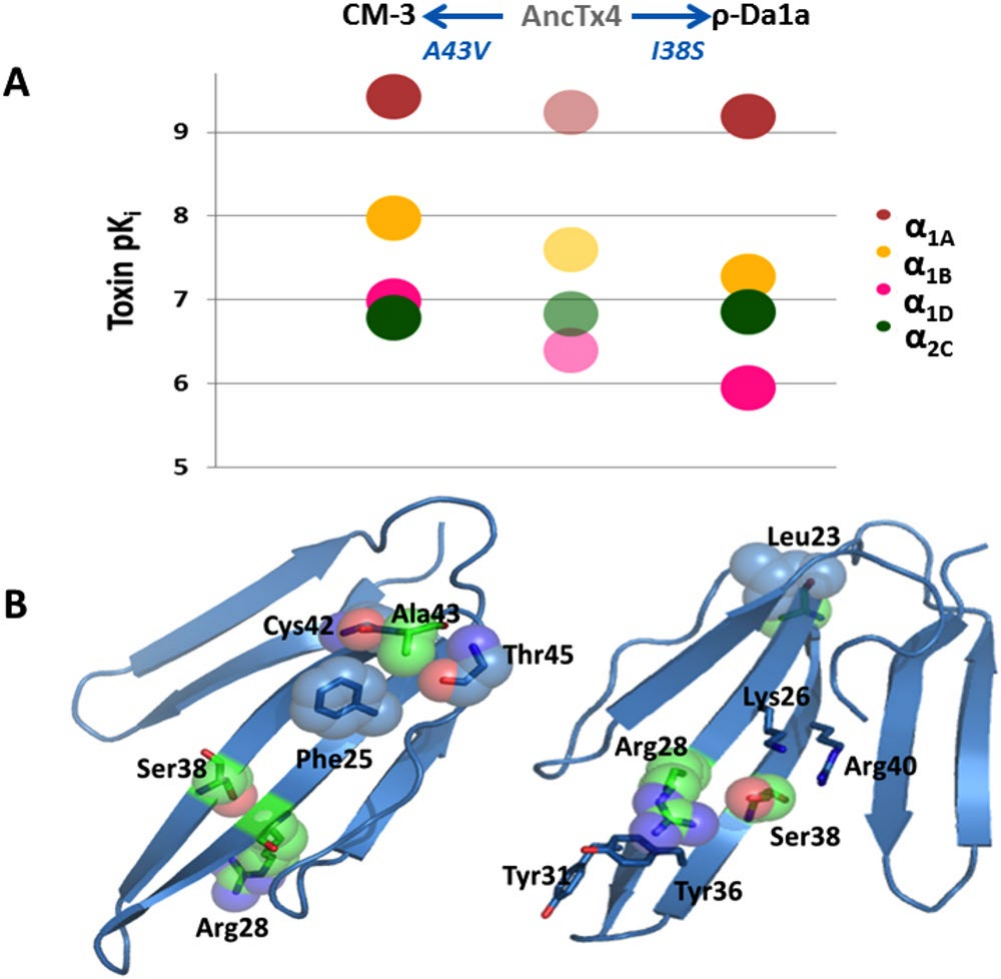
Affinity and structure analysis of CM-3, AncTx4 and ρ-Da1a toxins on adrenoceptors. (**A**) pK_i_ values are reported for CM-3, AncTx4 and ρ-Da1a on the 4 adrenoceptors α_1A_, α_1B_, α_1D_ and α_2C_. (**B**) The X-ray structure of ρ-Da1a K34A (code PDB: 4IYE), the position 43 is in the top of the third loop quite far and on the opposite side compare to the substitution S38I. The lateral chain of Ala43 is surrounded by the phenyl ring of Phe25, the side chain of Leu23, and by oxygen atoms from Cys42 and Thr45 main chains. This region of the globular core is indeed compact. Hence the substitution A43V has to reorganize the packing to accommodate the bigger lateral chain of the valine.

To understand the structure-activity relationship, the binding modulations that occurred between ancient and extant toxins for some receptor subtypes, needs to be analyzed. Since only a few substitutions occurred between each toxin (from AncTx2 to MTβ, see Fig. 3), inferring the structure-activity relationships (SAR) becomes possible. MTβ/CM-3 displayed similar pharmacological profiles on their four GPCR targets relative to ρ-Da1a, except for a slight improvement in affinity toward α_1B_ and α_1D_ subtypes by 5 and 10-fold, respectively (Table 1, Fig. 5). The double substitution S38I-A43V between ρ-Da1a and CM-3/MTβ might be responsible for these differences. AncTx4 showed an intermediate sequence between CM-3/MTβ and ρ-Da1a, with only one substitution (S38I or V43A) compared with these two modern toxins. AncTx4 can be positioned in term of affinity for adrenoceptors between CM-3/MTβ and ρ-Da1a (Fig. 6A), suggesting. that these 2 substitutions (S38I and V43A) contributed to the 5–10 fold affinity increase for the α_1B_ and α_1D_ receptors. The evolutionary pattern illustrates a slight drop in affinity between the α_1B_ and α_1D_ receptors and CM-3, AncTx4 and ρ-Da1a, but not for the α_1A_ and α_2C_ subtypes where the pK_i_ remained constant (Fig. 6A). As shown on the X-ray structure of ρ-Da1a (code PDB: 4IYE, Fig. 6B), position 43 is located at the top of the toxin, at the beginning of the third loop, far away and on the opposite side to position 38 located in the middle of the loop II.

#### Ancestral resurrection and engineering opportunities

**Figure 7:**
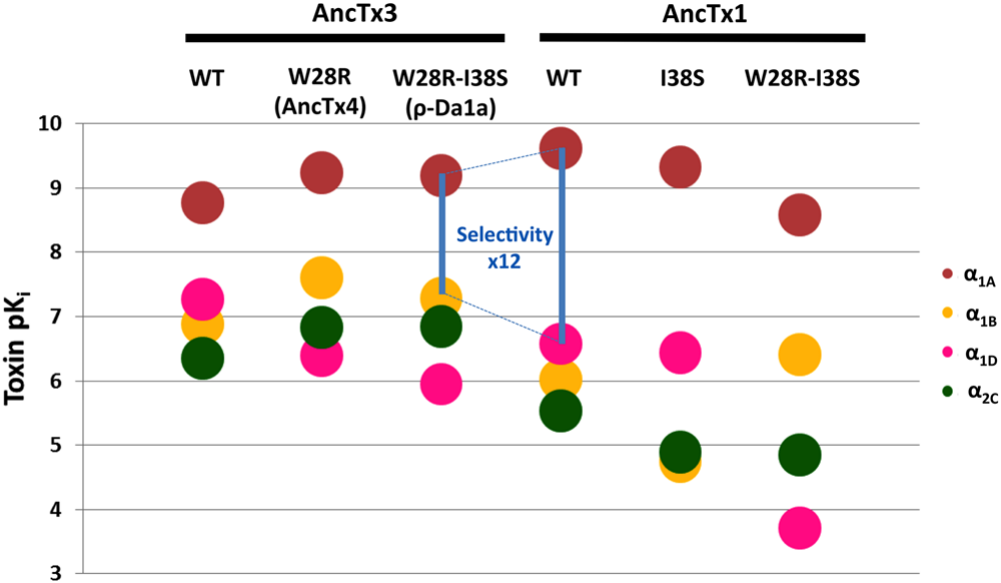
Affinity constants of AncTx1 and AncTx3 variants on adrenoceptors. pK_i_ values are reported for AncTx3, AncTx4, ρ-Da1a, AncTx1 and its 2 variants (AncTx1 I38S and W28R-I38S) on the 4 adrenoceptors α_1A_, α_1B_, α_1D_ and α_2C_. The 12-times increase in selectivity for α_1A_ between ρ-Da1a and AncTx1 is shown. AncTx4 and ρ-Da1a are here represented as AncTx3 variants (W28R and W28R-I38S respectively), like I38S and W28R-I38S for AncTx1, in order to compare the effect of these substitutions in AncTx3 and AncTx1.

A SAR for the α_1_ receptor subtypes was discerned from the analysis of the effect of the substitutions that differentiate AncTx3 from ρ-Da1a, which affect their respective pharmacological profiles. The substitution in AncTx4 (W28R) and those in ρ-Da1a (W28R + I38S) cause a substantial loss in affinity for α_1D_ receptor (pK_i_ dropping from 7.27 to 5.95) (Fig. 7). The selectivity profile of AncTx1 for the α_1A_ subtype surpasses that of all modern and ancestral aminergic toxins (Figs 5 and 7, Table 1) with a selectivity factor (the ratio between the affinity constants for the 2 most highly targeted receptors) that is 12-times better in AncTx1 compared to ρ-Da1a, currently the most selective peptide for the α_1A_ subtype^35^. The selectivity of AncTx1 is characterized by a slightly higher affinity for α_1A_ and a much lower one for the α_1B_ and α_2C_ receptors, relative to ρ-Da1a (Fig. 7). The selectivity of AncTx1 for the α_1A_ relative to the α_1D_ subtype suffers a small setback compared to ρ-Da1a (pK_i_ 6.58 for AncTx1, 5.95 for ρ-Da1a). Having recognized that the substitution (I38S) in AncTx3 affects α_1D_ binding and that (W28R) increases α_1B_ and α_2C_ binding, the appropriate substitutions were integrated stepwise in AncTx1 to boost its selectivity. The selectivity profile for the variant AncTx1 I38S is characterized by pK_i_ values of 9.33, 4.75, 6.44 and 4.90 for α_1A_, α_1B_, α_1D_ and α_2C_ subtypes, respectively (Fig. 7, Table 1). These values attest to a drop in affinity of AncTx1 I38S for α_1B_ and α_2C_ compared to AncTx1, but not for the α_1D_ subtype for which the pK_i_ hardly changed (6.44 vs. 6.58 for AncTx1). The pK_i_ values for the AncTx1 double variant W28R-I38S were calculated to be 8.59, 6.41, 3.71 and 4.85 toward α_1A_, α_1B_, α_1D_ and α_2C_ respectively (Fig. 7, Table 1) showing that despite an impressive loss in α_1D_ affinity (3.71 vs. 6.58 for AncTx1), the selectivity of this variant suffered from a large pK_i_ gain toward α_1B_ (6.41 vs. 4.75 for AncTx1 I38S). While ancestral toxin resurrection gave valuable binders with novel characteristics and pointed out key substitutions that can modulate aminergic toxins binding properties, combining this knowledge together in a rational manner to refine the pharmacological characteristics, ran into problems typical of classical rational and semi-rational protein engineering projects.

### MT3-MT1 pathway

**Figure 8:**
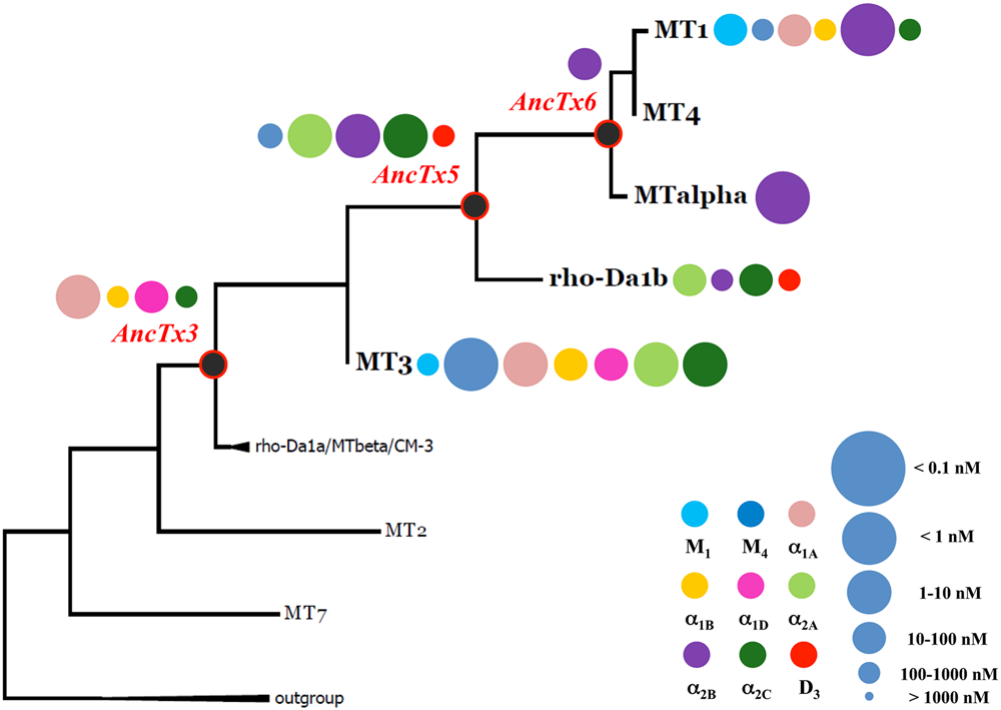
Maximum likelihood tree and associated function of the aminergic toxins from MT3-MT1 evolutionary pathway. Bootstraps value higher than 50% are shown. Toxin’s functions are illustrated by circles identifying the receptor targeted (color) and the corresponding K_i_ value (circle size), as described in the right legend, at the right side of each toxin name.

The second branch of the evolutionary tree comprises toxins that are more functionally divergent (compare ρ-Da1a/CM-3/MTβ to MT3 and MT1 in Fig. 1B). While MTα is exquisitely selective for the α_2B_ receptor, MT3 has the most divergent profile with an activity that spans seven of the nine receptors studied. MT3 interacts with the adrenoceptors α_1A_, α_1B_, α_1D_ and α_2C_ subtypes with comparable pK_i_ values (Fig. 8, Table 2). AncTx3 shared such an activity with notable differences with respect to α_2C_, for which MT3 has gained an affinity that is two orders of magnitude higher (pK_i_ = 8.82 vs 6.35 for AncTx3). MT3 recognized three extra targets compared to AncTx3, namely the α_2A_ adrenoceptor and the muscarinic M_1_ and M_4_ subtypes with pK_i_ values ranging from 6.22 for M_1_ to 9.08 for M_4_ (Fig. 8). The 10 substitutions that distinguish MT3 from AncTx3, responsible for these selectivity differences, are located exclusively on fingers I and II (Fig. 3). The branching out of AncTx5 from MT3 was associated with a drastic modification of the pharmacological profile (Fig. 8). AncTx5 preserved only the interactions with the α_2A_ and α_2C_ adrenoceptors, while those with M_1_, α_1A_, α_1B_ and α_1D_ receptor subtypes were lost and the affinity for M_4_ suffered a massive decrease (pK_i_ = 6.81 vs. 9.08 for MT3). However, AncTx5 recognizes the dopamine D_3_ receptor, a subfamily of receptor targeted until now by only one aminergic toxin^36^. The pK_i_ value of AncTx5 for this subtype was slightly higher (6.81) than for ρ-Da1b (6.12) (Table 2, Supplementary Fig. S3).

**Table 2:** Affinity constant (pKi ± SEM) of MT3, ρ-Da1b and AncTx5 toxins for muscarinic (M1, M4), adrenergic (α_1A_, α_1B_, α_1D_, α_2A_, α_2CB_, α_2C_) and dopaminergic (D3) receptors.

Toxin Receptor subtype	MT3^a^	AncTx5	ρ-Da1b^a^
α_1A_	8.84	<6	<6
α_1B_	7.68	<6	<6
α_1D_	7.88	<6	<6
α_2A_	8.51	8,33 ± 0,04	7.85
α_2B_	<6	8,11 ± 0,07	6.66
α_2C_	8.82	8,46 ± 0,01	7.44
M1	6.22	<6	<6
M4	9.08	6,81 ± 0,15	<6
D3	<6	6,81 ± 0,09	6.12

a: [19].

ρ-Da1b was first described for its interaction with the three α2 adrenoceptors with pK_i_ values of 7.85, 6.66 and 7.44 for α_2A_, α_2B_ and α_2C_ respectively^17^. Nevertheless, AncTx5 with pK_i_ values of 8.33, 8.11 and 8.46 on these same three subtypes was also, as for D_3_, a stronger binder than ρ-Da1b (Table 2). These higher pK_i_ values arise from the only five substitutions that distinguish these two toxins. These include charge modifications in finger I (N7D, E15Q) and modifications at positions 26 (K to I) and 28 (W to R), already shown as key determinants for target modulation^36^.

Due to the high number of substitutions at the other nodes (10 with an insertion at the AncTx5 node, see Figs 1A and 3), understanding the SAR was more difficult. Since finger I is identical in both MT3 and AncTx5, differences in target recognition as noted for M_4_, α_1A_, α_1B_, α_1D_, α_2B_ and D_3_ may be derived from the changes in fingers II and III of these two proteins. As differences between AncTx3 and MT3 come from fingers I and II, we hypothesized that variations in affinity noted for M_4_, α_2A_, or α_2C_ can be mapped to changes in these fingers.

AncTx6 represents the node that leads to MTα and MT1, two toxins that recently were shown to have a completely different pharmacological profile despite a 94% sequence identity^18^. MTα is active on only one of the six targets of MT1, the subtype α_2B_. Three substitutions in finger II (I31L, V32N and P33H) from MT1 to MTα are thought to be responsible for these functional differences. A single substitution marks the transition from AncTx6 to MTα. The parent shared the adrenoceptor α_2B_ as its only target (Figs 3 and 8). Considering the nature of the mutation, L31I, even a 10-fold difference in pK_i_ from AncTx6 to MTα (7.95 vs 9.05) was unexpected.

## Discussion

In the last decades, directed evolution was largely exploited as a protein engineering strategy to mimic natural protein evolution by an iterative process combining the generation of molecular diversity by random mutagenesis and the high throughput screening selection of library members with improved functions^37^. This approach was recently applied using cDNA display on a three-finger scaffold, in order to produce novel molecules such as interleukin-6 receptor ligands^21^ and serine protease inhibitors^22^. Nevertheless, in order to obtain smaller but functionally rich libraries, rational protein engineering was developed by exploiting information on protein sequence, structure and function^29^. We applied this strategy on aminergic toxins using a loop permutation approach and create chimeric toxins with new functional profiles on muscarinic and adrenergic receptors^23^. Nevertheless, within a protein family with many functionally diverse members, a vertical strategy which reconstruct ancestral genes and study the effect of mutations on functional diversification should allow the identification of the structural determinants of protein function and the exploitation of this knowledge for protein engineering^24^.

For the first time on animal venom toxins, we were able to apply phylogenetic analysis to resurrect ancestral proteins and obtain new and unique SAR. The aminergic toxin family is composed of closely related toxin isoforms with diverse pharmacological profiles on the biogenous amine GPCR, meaning that few substitutions can trigger important functional changes. Thus, this toxin family provided a suitable model for ancestral protein resurrection studies. Given that the phylogenetic reconstructions were sufficiently robust with very few ambiguous ancestral sites (8% under the symbolic P < 0.9), we retained only six ancestral toxins (AncTx) with unique and intermediate primary structures relative to extant aminergic toxins. Interestingly, most of them also showed subtle and profound modulations in their pharmacological profiles. All these changes could then be directly correlated to amino acids substitutions. We also found three key positions in the ρ-Da1a evolutionary pathway (positions 28, 38 and 43) that can modulate by varying degrees the affinities for the α_1A_, α_1B_, α_1D_ and α_2C_ subtypes. Based on the AncTx4 pharmacological profile, the complementary role of positions 38 and 43 in the pharmacological selectivity of these toxins was reinforced to confirm previous conclusions made for ρ-Da1a, CM-3 and MTβ aminergic toxins^36^. Given that position 43 is located in the globular core of the toxin, a direct involvement in receptor interaction is unlikely. The reason for its impact is unclear but it may involve subtle structural effects. Interestingly, this region of 3FT has already been highlighted in a directed evolution work^21^. Despite a complete “randomization” of the tips of the three loops (23 amino acids out of 61), an additional mutation at position 44 occurred spontaneously in their most represented population of interleukin 6 receptor binders^21^. Regarding position 28, for the first time it was revealed crucial in adrenoceptors binding. This position is proximal to residue 38, confirming this region as key for adrenergic receptor binding. The mechanism behind the binding modulation at position 28 needs to be clarified but it might be due to a stabilizing effect at the tip of finger II as suggested for position 38 (ref. 36). In the MT3-MT1 pathway, despite the sequence variability between ancestral and extant toxins, the important positions for the strong affinity for α2 adrenoceptors were determined (positions 7 and 15 in finger I and 26 and 28 in finger II).

On the basis of these freshly obtained SAR, new toxins variants with desired properties were engineered. We increased the affinity of AncTx1 for the α_1B_, α_1D_ or α_2C_ receptors with just two variants, unfortunately without achieving our primary goal to improve selectivity. Epistasis, a phenomenon already stated as important in protein evolution may have been the cause as revealed by other ancestral resurrection studies^27^. Indeed, the functional effects of a substitution could depend on its surrounding context or sequence background. Despite close homology, the same substitutions introduced in AncTx1 or AncTx3 did not result in either a decrease nor an increase in affinity for receptors α_1A_, α_1B_ and α_1D_.

In this study of ancestral toxins, we have obtained new, original and potentially useful functional profiles. AncTx1 is now the most selective peptide known for the α_1A_ adrenoceptor subtypes. AncTx5 is the most potent aminergic toxin able to interact with the three α2 adrenoceptor subtypes. The recognition mechanism and binding mode of this toxin toward the three receptor subtypes become of great interest.

## Conclusion

Our study contributes to the validation of the ancestral protein resurrection methodology that uses a small but functionally rich toxin library. Most of our results come from the first evolutionary pathway related to ρ-Da1a. Nonetheless, the second pathway opens a new promising line of investigation to understand the structure-activity relationships by delineating a few important positions.

The aminergic toxins have proved to be good candidates for ancestral protein resurrection methodology as a small number of variants generated diverse pharmacological profiles. Undoubtedly, further studies will lead to methodology improvements derived from better evaluation of the sequence diversity and its link to the functional divergence before starting the resurrection project. The advantages over other protein engineering strategies are clear: a smaller number of variants with a more diverse activity profiles. Understanding the causes of the epistasis phenomenon would remove it as an obstacle and help future protein engineering work.

## Methods

### Materials

All tritiated radioactive ligand were purchased from PerkinElmer Life Sciences (Courtaboeuf, France). N-methylscopolamine, prazosin, yohimbine and spiperone were from Sigma-Aldrich (St Quentin-Fallavier, France). As previously described in Blanchet *et al*.^19^, membrane preparations were obtained from transient (COS cells) or stable (CHO cells) cell lines prepared in the laboratory.

### Peptide synthesis, disulfide bond formation and protein purification

As previously described in Blanchet *et al*.^36^, the six AncTxs and the two engineered AncTx1 variants were synthesized on a 50 µmol scale using Fmoc chemistry on a Prelude synthesizer (Protein Technologies®) using HMPB ChemMatrix® resin functionalized with the appropriate protected amino acid (AA). Peptides were purified by reverse phase semi-preparative HPLC using a Vydac C18 column (250 × 10 mm) with an optimized gradient (usually 40 to 60% of solvent B in 40 min (A: 0.1% TFA in H_2_O, B: 60% acetonitrile and 0.1% TFA in H_2_O). The peptides were then subjected to folding in a buffer Tris-HCL 100 mM pH 8 containing 1 mM of EDTA, 1 mM of GSH and GSSG and 20% v/v of glycerol. The final reticulated peptides were purified using the same method described above and characterized by ESI MS (Bruker Esquire 3000 plus).

### Circular dichroism analysis

CD spectra were recorded on a Jasco J-815 CD spectropolarimeter. As previously described in Blanchet *et al*.^36^, measurements were routinely performed at 20 °C in 0.1 cm path length quartz cells (Hellma) with a peptide concentration of 20.10^−6^ M in 5 mM sodium phosphate buffer pH 7.4. Spectra were recorded in the 190 to 260 nm wavelength range. Each graphic represents the average of three spectra.

### Crystallization and structure determination of AncTx1-W28R/I38S

Crystallization experiments were carried out with lyophilized toxin redissolved at 5 mg/ml in 1 M sodium acetate, pH 5.5. The crystallization trials were carried out using Cryst Chem^TM^ sitting drop vapor diffusion plates with 1 µl drops of protein and precipitant, stored in a constant temperature incubator at 20 °C. Limited screening for crystals was carried out starting from conditions used for MT7^33^. Crystals of AncTx1-W28R/I38S were obtained from 1.9 M ammonium sulfate, 4% MPD, 2% 1,4-dioxane, 2% gamma-valerolactone, 0.132 M sodium citrate, pH 5.5. Four crystals were flash frozen in a cryoloop with a cryoprotectant consisting of 80% saturated lithium sulfate, 10% dioxane and 10% gamma-valerolactone. The crystals were shipped for automatic data collection at the Massif 1 at the ESRF in Grenoble, France on beamline ID30A-1^38^. The XDS^39^ was used for data reduction with the script XDSME (https://github.com/legrandp/xdsme). The crystals belonging to the trigonal space-group P3_1_21 with cell parameters a = b = 41.1 c = 67.1 Å diffracted to between 1.95 and 1.8 Å resolution. The structure was solved by molecular replacement starting from the ρ-Da1a crystal structure (PDB ID: 4IYE)^34^ with Phaser^40^. Refinement was carried out with REFMAC^41^ and Phenix^42^ and the electron density fitted with COOT^43^. Statistics for AncTx1-W28R/I38S structure are reported in Table S1.

### Binding experiments

The six AncTxs were first screened on the 12 previously identified aminergic receptor targets reported for the aminergic toxins (namely M_1_ to M_5_ muscarinic receptor subtypes, all three α1 and α2 adrenoceptor subtypes and the dopamine D_3_ subtype) by heterologous inhibition experiment as described in Blanchet *et al*.^19^. In short, 5 µM of each AncTxs were incubated (in duplicate) with a fixed amount of membrane preparation and a fixed concentration of a specific radioligand in 96-wells plate with 100 µl as final volume for 20h at RT. As previously described in Blanchet *et al*.^19^, binding experiments for muscarinic receptor subtypes, were carried out in saline phosphate buffer (10 mM sodium phosphate, 135 mM NaCl, 2.5 mM KCl), pH 7.4, with 0.1% bovine serum albumin (BSA). [^3^H]-NMS was used as specific radioligand at 1 nM final concentration. The adrenergic α_1_ membranes were incubated in Tris buffer (50 mM TrisHCl, 10 mM MgCl_2_), pH 7.4, 0.1% BSA, with 1.6 nM of [^3^H]-Prazosin as radiotracer. For α_2_ adrenoceptors, binding was carried out in saline HEPES buffer (50 mM HEPES, 5 mM MgCl_2_, 1 mM CaCl_2_), pH 7.4, 0.1% BSA and 1.7 nM of [^3^H]-Rauwolscine. Finally, binding assays on D_3_ dopamine receptor subtype were carried out in Tris buffer (50 mM TrisHCl, 10 mM MgCl2), pH 7.4, 0.1% BSA with 2 nM of [^3^H]-Methylspiperone. The affinity constants of the radioactive ligands for each membrane preparations were determined by equilibrium saturation experiments (n ≥ 2). Non-specific binding values were obtained by using N-methylscopolamine, prazosin, yohimbine and spiperone in excess (concentration up to 1000 times their K_d_) in the binding assay on respectively: the muscarinic, α_1_, α_2_ and D_3_ receptor subtype membrane preparations. Binding reactions were stopped by filtration through GF/C filter pre-soaked in 0.5% polyethyleneimine on a cell harvester (PerkinElmer, Courtaboeuf, France) and plates were dried. Ultimagold O (25 ml, PerkinElmer) was added to each well and samples were counted using a Top-Count counter (PerkinElmer, Courtaboeuf, France). After subtraction of the non-specific binding and normalization, only radiotracer displacement of 20% or more was considered as relevant and subjected to competition experiments.

AncTxs able to inhibit the radioligand at 5 µM were then subjected to dose-response competition experiments. In this experiment, various concentrations of AncTx were used in order to determine their inhibitory constant. This experiment followed exactly the same procedure described earlier. The binding data from individual experiments (n ≥ 2) were analyzed by non-linear regression analysis using Kaleidagraph 4.0 (Synergy software, Reading, PA, USA). After subtraction of the non-specific binding and normalization, a one-site inhibition curve was fitted to inhibition binding data, IC_50_ values were converted to K_i_ using the Cheng-Prusoff equation^44^.

### Phylogenetic reconstruction and resurrection

As described previously in Blanchet *et al*.^19^, sequences of aminergic three-finger toxins were retrieved from the UniProt Knowledgebase. Sequence alignments were carried out using MUSCLE from the MEGA5 package^45^. Alignments were visualized and inspected manually for eventual errors with the MEGA5 alignment visualizer and Jalview version 2^46^ was used to edit the final colored alignment. The maximum likelihood method^47^ was used to reconstruct the phylogenetic trees. The best-fit substitution model, tree reconstruction, visualization, and annotation were chosen using MEGA5. Bootstraps support values were based on 10 000 replicates. Each ancestral amino acid and its associated probability value (P) at every site of each node were retrieved from an excel file supplied by MEGA5 and used to reconstruct the most probable ancestral sequence for each node of the tree. Few ambiguous sites were found, and to minimize the number of possible sequences, the amino-acid with the highest P was every time chosen.

The position 38, located at the middle of the second loop, is quite closed to the tip of the loop II (known as a common “hot spot” for 3FTx to interact on their various targets) and may affect close and functionally important residues. Furthermore, as previously described, the S38I substitution could lead to a local stabilization of the Arg28, Arg40, Lys26 and Tyr36 lateral chains.

## Electronic supplementary material

Supplementary information
