# Supplementary material for: Ancestral protein resurrection and engineering opportunities of the mamba aminergic toxins
Source: Sci Rep. 2017 Jun 2;7:2701. doi: 10.1038/s41598-017-02953-0 (PMC5457417; doi:10.1038/s41598-017-02953-0)
Supplement: Supplementary file 1 — Supplementary information [file 41598_2017_2953_MOESM1_ESM.pdf]

Supplemental Information for:

Ancestral protein resurrection and engineering opportunities of the mamba aminergic toxins

Guillaume Blanchet[1, 2], Doria Alili[1], Adèle Protte[1], Gregory Upert[1], Nicolas Gilles[1], Livia Tepshi[1], Enrico A. Stura[1], Gilles Mourier[1], and Denis Servent[1*]
[1]CEA, Institut des Sciences du Vivant Frédéric Joliot, Service d'Ingénierie Moléculaire des Protéines (SIMOPRO), Gif-sur-Yvette, 91190, France. [2]UFR Sciences de la Vie, Université Pierre et Marie Curie (UPMC), 4 place Jussieu, Paris, 75005, France.
Correspondence should be addressed to D.S: denis.servent@cea.fr

Table of Contents:

Table S1 ....page 2
Figure S1 ....page 3
Figure S2 ....page 4
Figure S3 ....page 5

Table S1: Statistics for AncTx1-W28R/I38S :
Data collection, processing and refinement

**PDB code**	**5MG9**
**Data Collection**	
Synchrotron source	ESRF, Massif-1 ID30A-1
Wavelength (Å)	0.965
Space group	P3 1 21
Unit-cell parameters (Å)	41.1 41.1 67.1
Molec./asym.	
Resolution (Å)	35.6-1.8 (1.91-1.80)
R meas (%)	11.2 (161.5)
R factor (%)	7.8 (152.4)
Mean I/ σ (I)	13.05 (1.21)
CC(1/2) (%)	99.9 (75.6)
Completeness (%)	100 (100)
Multiplicity	9.34 (9.21)
**Refinement**	
Resolution (Å)	35.6-1.80 (1.91-1.80)
No. of reflections	11685 (1785)
No. of reflections (non-anomalous)	6410
R_{work} (%)	20.32 (38.69)
R_{free} (%)	23.65 (41.17)
**r.m.s. deviations**	
Bond lengths (Å)	0.026
Bond angles (°)	7.679
**Ramachandran**	
favoured (%)	98.4
outliers (%)	0

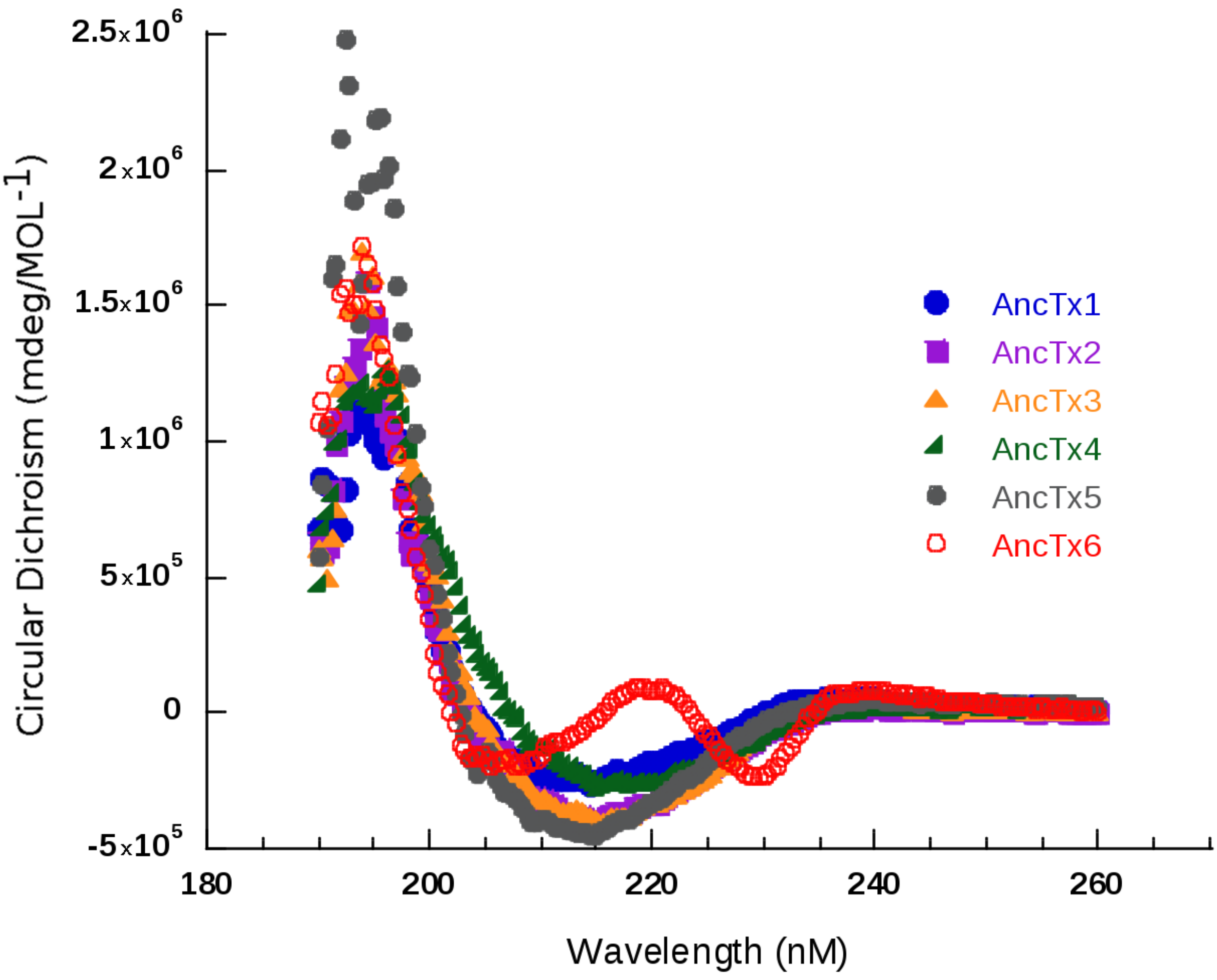

Suppl. Fig. S1. Far-ultraviolet CD spectra of the different ancestral toxins.
The spectra were recorded in water at 20°C at a peptide concentration of 5 µM

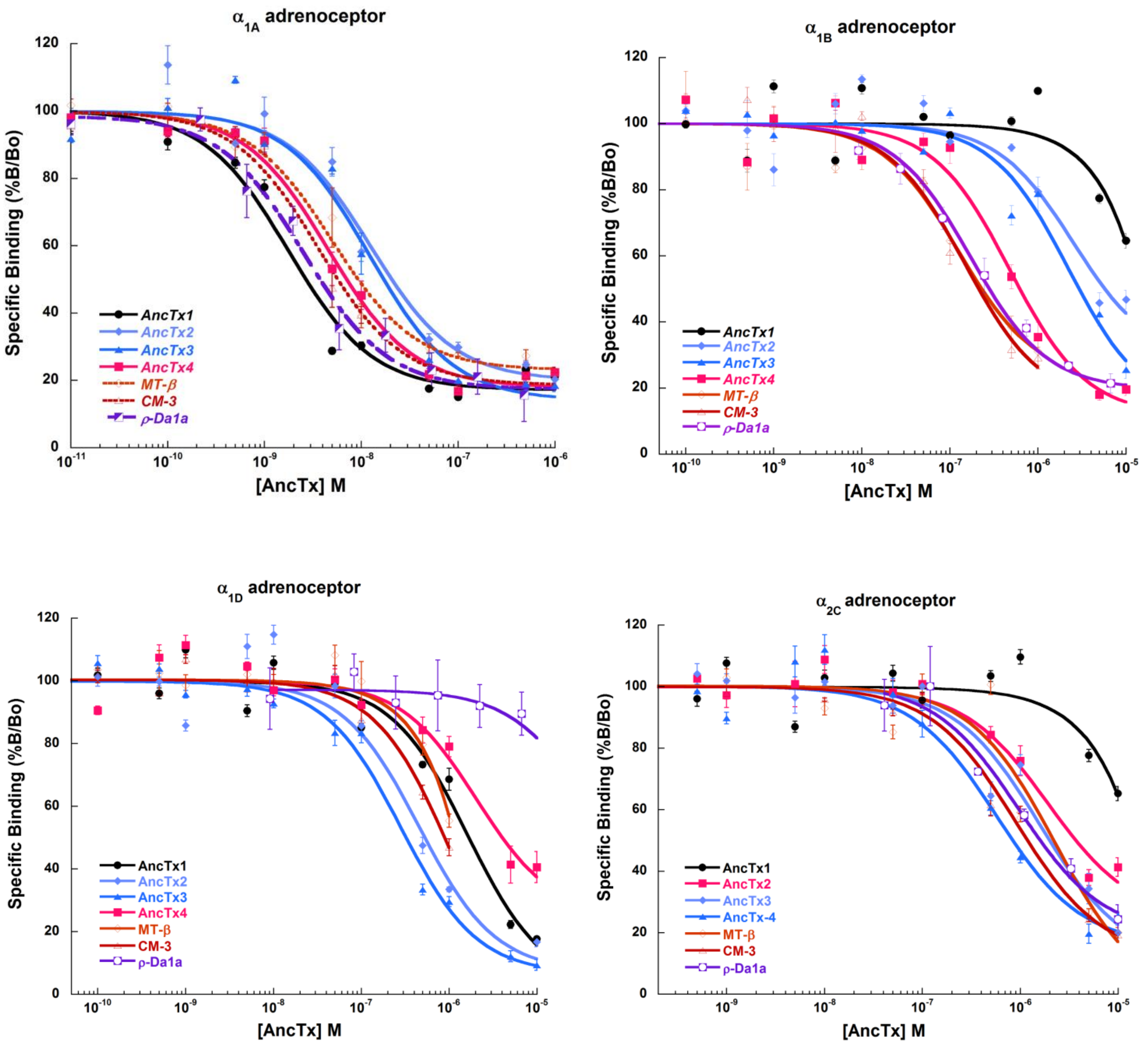

Suppl. Fig. S2. Inhibition of ^{3}H-Prazosin binding on various α_1-adrenoceptors and ^{3}H-rauwolscine on α_{2C} subtype by by natural and ancestral toxins

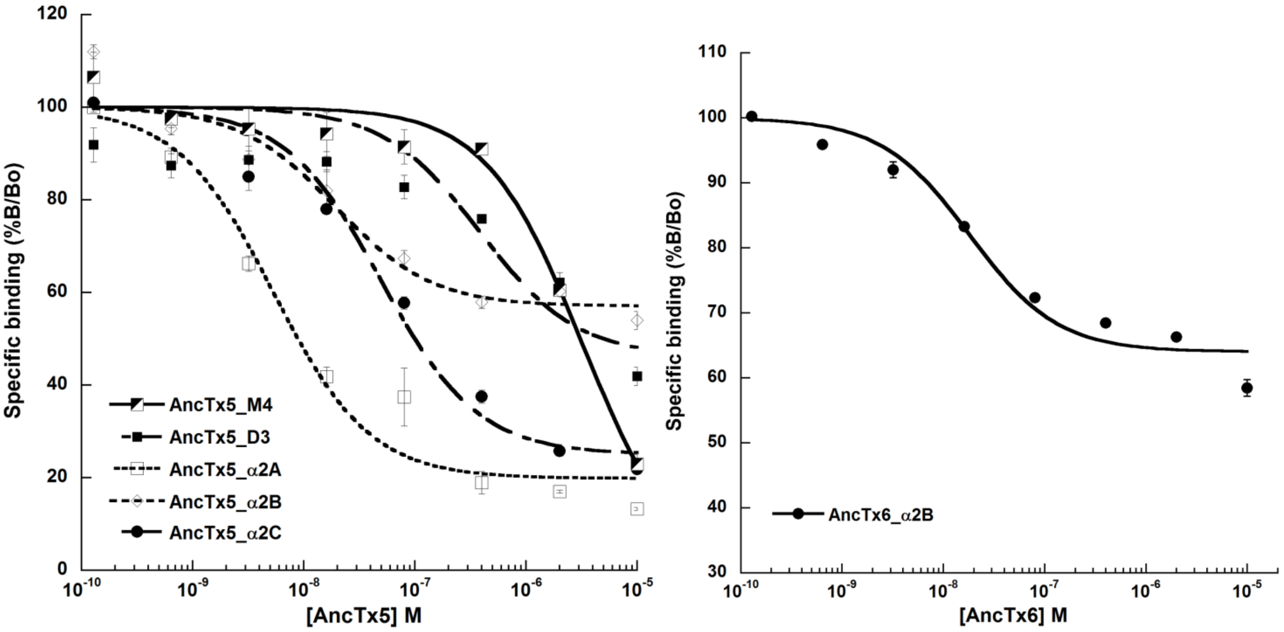

Suppl. Fig. S3. Inhibition binding curves of AncTx5 on α_2-adrenoceptors, dopamine D_3 and muscarinic M_4 receptors (left). Inhibition binding curves of AncTx6 on α_{2B}-adrenoceptor (right).
